# Transcriptome and Regional Association Analyses Reveal the Effects of Oleosin Genes on the Accumulation of Oil Content in *Brassica napus*

**DOI:** 10.3390/plants11223140

**Published:** 2022-11-16

**Authors:** Yuan Jia, Min Yao, Xin He, Xinghua Xiong, Mei Guan, Zhongsong Liu, Chunyun Guan, Lunwen Qian

**Affiliations:** Collaborative Innovation Center of Grain and Oil Crops in South China, Hunan Agricultural University, Changsha 410128, China

**Keywords:** oleosin, *Brassica napus*, seed oil content, co-expression

## Abstract

Rapeseed stores lipids in the form of oil bodies. Oil bodies in the seeds of higher plants are surrounded by oleosins. Adjusting oleosin protein levels can prevent the fusion of oil bodies and maintain oil body size during seed development. However, oil contents are affected by many factors, and studies on the complex molecular regulatory mechanisms underlying the variations in seed oil contents of *B. napus* are limited. In this study, a total of 53 *BnOLEO* (*B. napus* oleosin) genes were identified in the genome of *B. napus* through a genome-wide analysis. The promoter sequences of oleosin genes consisted of various light-, hormone-, and stress-related cis-acting elements, along with transcription factor (TF) binding sites, for 25 TF families in 53 *BnOLEO* genes. The differentially expressed oleosin genes between two high- and two low-oil-content accessions were explored. *BnOLEO3-C09*, *BnOLEO4-A02*, *BnOLEO4-A09*, *BnOLEO2-C04*, *BnOLEO1-C01,* and *BnOLEO7-A03* showed higher expressions in the high-oil-content accessions than in low-oil-content accessions, at 25, 35, and 45 days after pollination (DAP) in two different environments. A regional association analysis of 50 re-sequenced rapeseed accessions was used to further analyze these six *BnOLEO* genes, and it revealed that the nucleotide variations in the *BnOLEO1-C01* and *BnOLEO7-A03* gene regions were related to the phenotypic variations in seed oil content. Moreover, a co-expression network analysis revealed that the *BnOLEO* genes were directly linked to lipid/fatty acid metabolism, TF, lipid transport, and carbohydrate genes, thus forming a molecular network involved in seed oil accumulation. These favorable haplotypes can be utilized in molecular marker-assisted selection in order to further improve seed oil contents in rapeseed.

## 1. Introduction

Rapeseed (*Brassica napus* L., AACC, 2*n* = 38) is one of the most important oil crops, providing approximately 15% of vegetable oil worldwide [1]. It originated approximately 7500 years ago from a spontaneous hybridization between *B. rapa* (AA, 2*n* = 20) and *B. oleracea* (CC, 2*n* = 18) [2]. The large amounts of unsaturated fatty acids (FAs) present in rapeseed oil have made it widely accepted as vegetable oil for human consumption and as biofuel for industry [3]. Enhancing seed oil content (SOC) and oil production per unit area of land is of paramount importance to meet the growing demand for oilseed breeding programs [4]. SOC is a highly complex trait due to multiple genes that regulate various seed-storage-oil metabolisms [5]. The biological and metabolic pathways for triacylglycerol (TAG) synthesis have been well recorded in various studies [6,7].

In the past few decades, oil content-associated quantitative trait loci (QTLs) have been identified in nearly all 19 linkage groups, and vary from 3 to 27 QTLs in each chromosome by biparental linkage mapping [8,9,10,11,12,13,14,15]. Recently, genome-wide association studies (GWAS) have been widely used to dissect the regulatory loci and genetic architecture of SOC at the whole-genome level. For example, a genome-wide association study conducted by Liu et al. [16] identified 50 loci that were significantly associated with seed oil contents in 521 rapeseed accessions. GWAS was used to detect the associations of 112 single nucleotide polymorphisms (SNPs) with seed quality traits in 405 *B. napus* inbred lines [17]. Tang et al. [18] used GWAS to detect the significant associations of 27 QTLs with SOC in different environments for 500 *B. napus* inbred lines. Xiao et al. [19] identified 17 loci that were significantly associated with seed oil content by GWAS, with 385,692 SNPs. GWAS was used to reveal nine haplotype regions that were significantly associated with the oil content in 203 Chinese semi-winter rapeseed accessions [20]. Although a large number of QTLs that affect oil content accumulations have been identified, their molecular mechanisms remain poorly understood.

With the development of sequencing and statistics, these approaches have been extensively applied to profile genome-wide gene expressions in crops. Weighted gene co-expression network analysis (WGCNA) based on transcriptome data has become one of the most efficient methods to display the correlations between genes in a pairwise manner to construct network graphs [21]. Clustering genes show similar expression patterns across many samples, as they could be members of the same pathway or biological process. These co-expression network properties can be further correlated with target traits to find the functional genes [22]. For example, Cui et al. [23] established an acyl-lipid metabolism co-expression network and detected 12 hub genes related to oil content accumulations. WGCNA was used to identify five modules that were highly associated with high-quality fiber, as well as five transcription factor (TF) genes playing an important role in fiber development [24]. A co-expression network was used to detect seven hub genes that were involved in soybean oil and seed storage protein accumulations [25].

Oleosins are hydrophobic plant proteins related to small (0.6–2 μm diameter) storage oil droplets, termed oil bodies [26]. At present, six oleosin lineages have been reported, including P, U, SL, SH, T, and M. The P (primitive) lineage is mainly distributed in green algae and may be the source of U (universal) oleosins, which further gives rise to the SL (seed low molecular weight) and SH (seed high molecular weight) lineages. In addition, the T (tapetum) lineage was detected only in the tapeta of Brassicaceae, and the M (mesocarp) lineage was detected in Lauraceae [27,28,29]. Oleosins are found in the oil bodies of seeds, tapetum cells, and pollen, but not in fruits [30]. Many studies have shown that adjusting oleosin protein levels can prevent the fusion of oil bodies and maintain oil body size during seed development [6,31,32]. Siloto et al. [33] suggested that a lack of oleosins caused fusion of oil bodies, leading to a decrease in oil content during the middle stage of seed development in *Arabidopsis thaliana*. Overexpression of *GmOLEO1* was found to increase the numbers of oil bodies and oil accumulations in soybeans [34].

In this study, the expression patterns of the oleosin genes in high- and low-oil-content rapeseed inbred lines were analyzed during different developmental stages of the seed. The novel loci of the oleosin genes that affect the accumulation of seed oil content were further identified by resequencing 50 rapeseed accessions with 532,005 SNPs. A co-expression network was also employed to reveal the regulatory network of oleosin genes in the accumulation of oil content. Together, our findings lay a foundation for an enhanced understanding of the role of oleosin genes in the accumulation of oil content in *B. napus*.

## 2. Results

### 2.1. Genome-Wide Identification of Oleosin Family Genes in the Genome of B. napus

Based on the hidden Markov model (HMM) and BLAST analysis, 16 and 53 oleosin genes were identified in Arabidopsis and *B. napus*, respectively. Information on gene names, IDs, chromosomal locations, and amino acid numbers is listed in Table S1. In the present study, in order to analyze the evolutionary relationships of oleosin genes, a neighbor-joining (NJ) phylogenetic tree was constructed based on 69 oleosin protein sequences that were divided into four lineages, namely, T, SL, SH, and U. The conserved motifs and exon and intron structures of the oleosin genes were then obtained (Figures S1 and S2). The T, SL, SH, and U lineages had 25, 8, 10, and 10 oleosin genes in *B. napus*, respectively. These results were consistent with the findings of Chen et al. [29].

### 2.2. Analysis of Cis-Acting Elements in Oleosin Genes of B. napus

**Figure 1:**
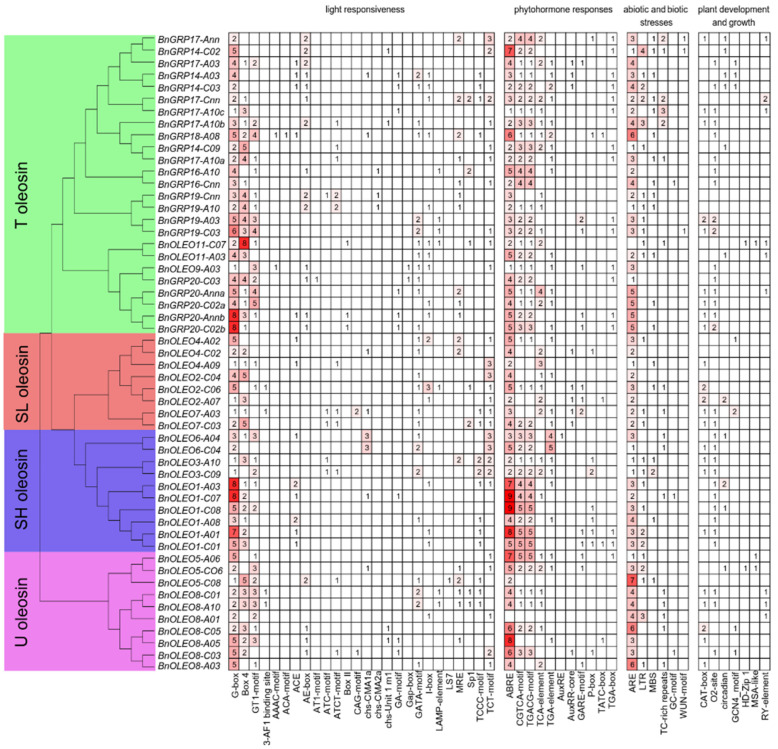
Analysis of cis-elements in the promoter regions of oleosin genes. The presence of different cis-acting elements was determined by the PlantCARE database.

The cis-elements in 53 *BnOLEO* (*B. napus* oleosin) genes were analyzed using the PlantCARE database. Cis-acting elements were found in the promoter region of the *BnOLEO* gene family, including elements primarily related to light, phytohormone, and abiotic stress responses (Figure 1, Table S2). The cis-regulatory elements included light-responsive elements, such as G-box, Box 4, and GT1-motif; phytohormone-responsive elements, such as ABRE (involved in the abscisic acid response); CGTCA- and TGACG-motifs (involved in methyl jasmonate response); TCA-elements (involved in salicylic acid response); motifs related to abiotic and biotic stresses, such as ARE elements (essential for anaerobic induction); TC-rich repeats (involved in defense and stress responses), LTR (involved in low-temperature responsiveness); and MBS (MYB binding site involved in drought-inducibility); (Figure 1, Table S2). There were many light-responsive elements in the promoters of *BnOLEO* genes, including the G-box identified in the promoters of 53 *BnOLEO* genes (with 3.50 on average for each promoter), Box 4 elements present in 39 of the 53 *BnOLEO* gene promoters (with 2.67 on average for each promoter), and GT1-motif elements present in 31 *BnOLEO* gene promoters (with 2.10 on average for each promoter). In addition, 212 ABREs associated with hormone response were unevenly scattered in the promoters of 52 *BnOLEO* genes, which were most enriched in the SH lineage (54/10) but rare in the T lineage (80/25). A total of 198 methyl jasmonate (MeJA) response elements showed relatively unbiased distributions in both the SH and T lineages. Additionally, 156 ARE elements were found to be common in the promoters of 49 *BnOLEO* genes (with 3.18 on average for each promoter; Figure 1, Table S2). These results indicated that oleosin played a role in regulating light, hormone, and abiotic stress responses.

### 2.3. Prediction of TF Binding Sites in BnOLEO Genes

**Figure 2:**
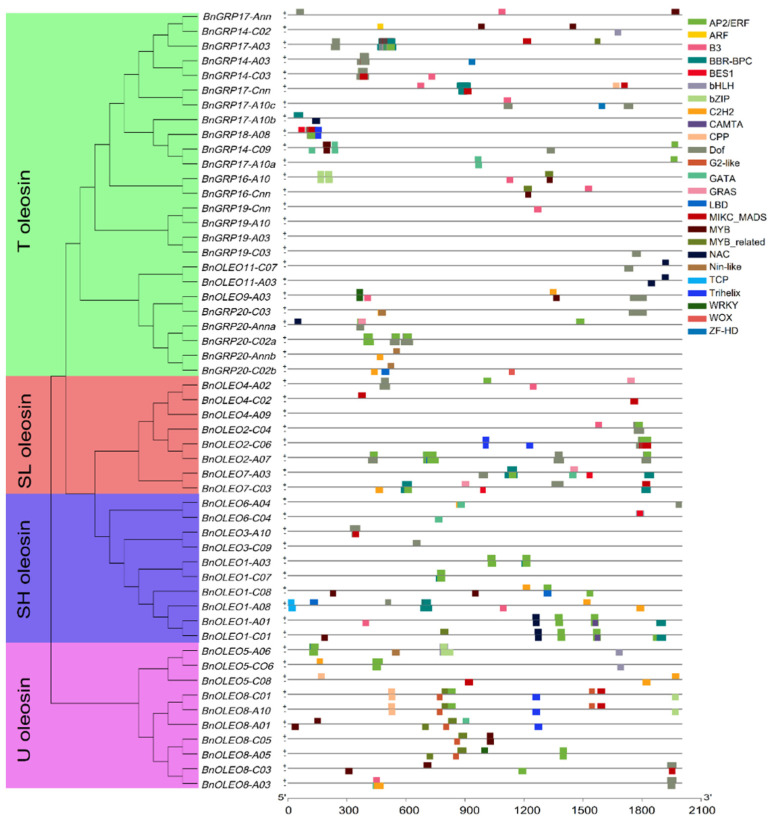
Transcription factor (TF) binding site analysis in the oleosin gene promoter. Boxes with different colors represent different transcription factor families. “+” and “−” represent positive and negative strands, respectively.

In order to investigate the regulation of TFs on the expressions of *BnOLEO* genes, the potential regulatory network of *BnOLEO* genes was inferred using PlantTFDB v5.0 (http://plantregmap.gao-lab.org/ (accessed on 10 May 2022)). The results showed that approximately 207 TFs from 25 TF gene families had potential target binding sites in the promoter region of *BnOLEO* genes (Figure 2, Table S3). The most enriched TFs belonged to the AP2/ERF superfamily containing the AP2/ERF domain (62 genes), WRKY DNA-binding protein (29 genes), basic leucine zipper (bZIP, 19 genes), DNA binding with one finger (Dof, 15 genes), and MYB (12 genes) families (Figure 2, Tables S3 and S4).

### 2.4. Expression Analysis of Oleosin Genes in B. napus with High and Low Oil Content

**Figure 3:**
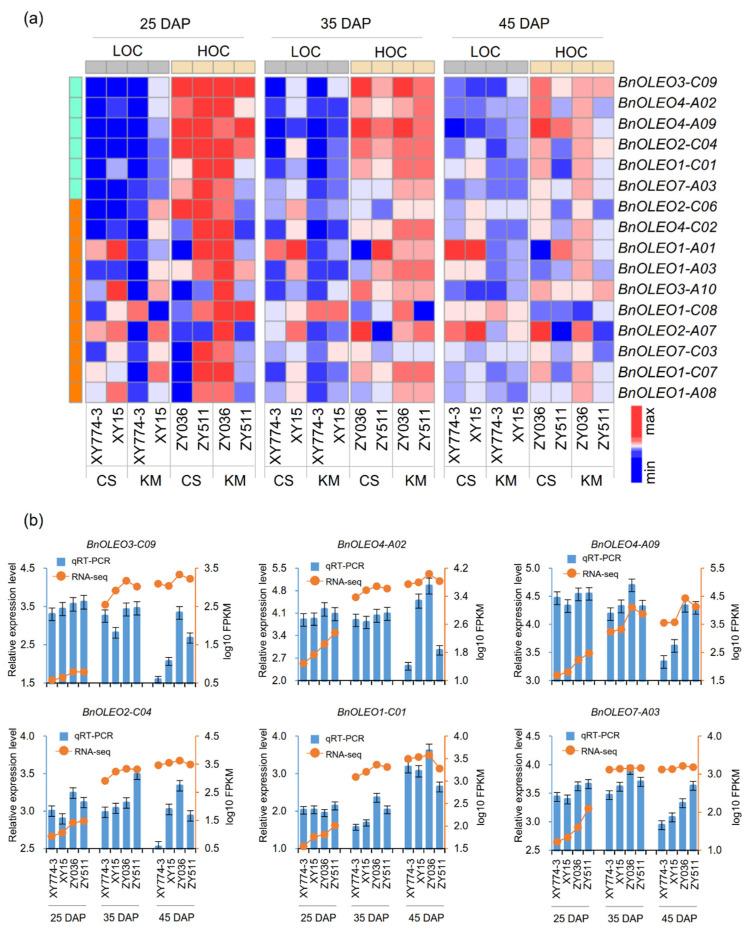
Expression patterns of oleosin genes at different stages of seed development after pollination in *B. napus*. (**a**) The heatmap is generated by comparing the relative results of different genes in different periods. Red, blue, and white indicate high, low, and medium expression levels, respectively. (**b**) qRT-PCR analysis of relative expression levels. The relative qRT-PCR expression levels (blue bar) are shown on the left *y*-axis. The RNA-Seq values (orange line) are shown on the right *y*-axis. *BnEF* was used as the reference gene. The relative transcript levels were averaged over the three technical replicates. LOC, low-oil content; HOC, high-oil content; CS, Changsha; KM, Kunming.

We investigated the expression of all oleosin genes in different tissues of Arabidopsis based on the Arabidopsis eFP Browser data (http://bar.utoronto.ca/efp/cgi-bin/efpWeb.cgi (accessed on 30 May 2022)) (Table S5). In Arabidopsis, the expression levels of oleosin genes in different lineages varied differently among different tissues. T lineage showed high expression levels in the flowering stage, but relatively low expression levels in other tissues. The expression levels of U, SL, and SH lineages gradually increased from seed stage 3 to 10 (exception for *AtOLEO3*), indicating that these lineages might be involved in seed development and oil content accumulation (Figure S3). Therefore, to investigate the expression patterns of oleosin genes in rapeseed, based on the gene expression database of two high-oil-content (HOC) and two low-oil-content (LOC) accessions, the expression profiles of *BnOLEO* genes at 25, 35, and 45 days after pollination (DAP) in two different environments were normalized to log_10_^FPKM^, and a heatmap was generated. The results showed that 16 oleosins belonging to the SH and SL lineages were expressed (Figure S4, Table S6). Among the 16 *BnOLEO* genes, *BnOLEO3-C09*, *BnOLEO4-A02*, *BnOLEO4-A09*, *BnOLEO2-C04*, *BnOLEO1-C01*, and *BnOLEO7-A03* showed higher expression in the HOC accessions than in the LOC accessions at 25, 35, and 45 DAP in two different environments (Figure 3a). In order to validate the transcriptional profiling results, quantitative real-time PCR (qRT-PCR) was performed to detect the transcript levels of 6 *BnOLEO* genes at 25, 35, and 45 DAP. The expression pattern of the genes obtained by qRT-PCR were consistent with the RNA-seq results, thereby confirming the accuracy of the RNA-seq data (Figure 3b).

### 2.5. Regional Association Analysis of Oleosin Genes

**Figure 4:**
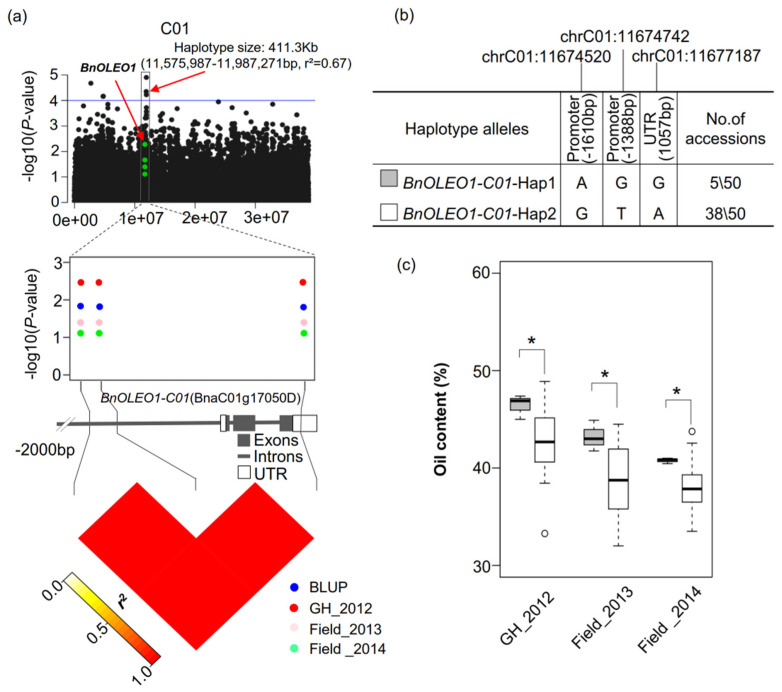
Regional association analysis of seed oil content on chromosome C01 by whole-genome resequencing of 50 accessions. (**a**) Haplotype region (11,575,987–11,987,271 bp, *r*^2^ = 0.67) was significantly associated with seed oil content. The chrC01:11,674,520, chrC01:11,674,742, and chrC01:11,677,187 SNPs are located in the promoter and UTR of *BnOLEO1-C01* (BnaC01g17050D) in the haplotype region, respectively. The heatmap shows that these SNPs have a strong LD. (**b**) Two haplotype alleles with frequencies greater than 0.01 were identified in *BnOLEO1-C01*. (**c**) Comparative analysis of two haplotype alleles related to the oil contents of inbred lines. The boxplots show that *BnOLEO1-C01*-Hap1 has a higher oil content than *BnOLEO1-C01*-Hap2. *: *p* ≤ 0.05.

Regional association analysis detected 182.4 Kb (13,459,752–13,642,146 bp, *r*^2^ = 0.71) and 411.3 Kb (11,575,987–11,987,271 bp, *r*^2^ = 0.67) haplotype regions that were significantly associated with oil content on the A03 and C01 chromosomes in the 50 re-sequenced rapeseed inbred lines, respectively (Figure 4a and Figure S5a). In the two haplotype regions, three SNPs were located in the *BnOLEO1-C01* and *BnOLEO7-A03* gene regions, which showed associations with SOC (Figure 4a and Figure S5a). Two and three haplotype alleles were identified in the *BnOLEO1-C01* and *BnOLEO7-A03* gene regions, respectively (Figure 4b and Figure S5b). Comparative analysis of the two and three haplotype alleles related to SOC revealed that *BnOLEO1-C01*-Hap1 and *BnOLEO7-A03*-Hap1 corresponded to accessions that showed higher SOCs compared to other haplotype alleles (Figure 4 and Figure S5, and Table S7).

### 2.6. Co-Expression Analysis of Oleosin Genes

In order to further analyze the function of oleosin genes, the transcriptome data of seeds at different developmental stages were used to construct co-expression networks. The analysis yielded 14 gene modules, each represented by a different color in the output (Figure S6a). *BnOLEO* genes were distributed in the blue and yellow modules (Figure S6c). The blue and yellow modules represent significant positive and negative correlations with high and low oil contents, respectively (Figure S6b). In order to provide a biological explanation of the gene network in the blue and yellow modules related to the *BnOLEO* genes, gene ontology (GO) enrichment analyses were conducted. In the blue module, in terms of the biological process ontology, the genes were mainly enriched for the ATP metabolic process (GO:0046034), ATP biosynthetic process (GO:0006754), and response to lipid (GO:0033993). For the molecular function ontology, the genes were mainly enriched for ATP binding (GO:0005524), carbohydrate derivative binding (GO:0097367), and lipase activator activity (GO:0060229; Figure S7 and Table S8), respectively. In the yellow module, the genes were mostly enriched for responses to high light intensity (GO:0009644), lipid storage (GO:0019915), and seed maturation (GO:0010431). For the cellular component ontology, the genes were mainly enriched for cytoplasm (GO:0005737) and lipid droplet (GO:0005811; Figure S7 and Table S8), respectively.

**Figure 5:**
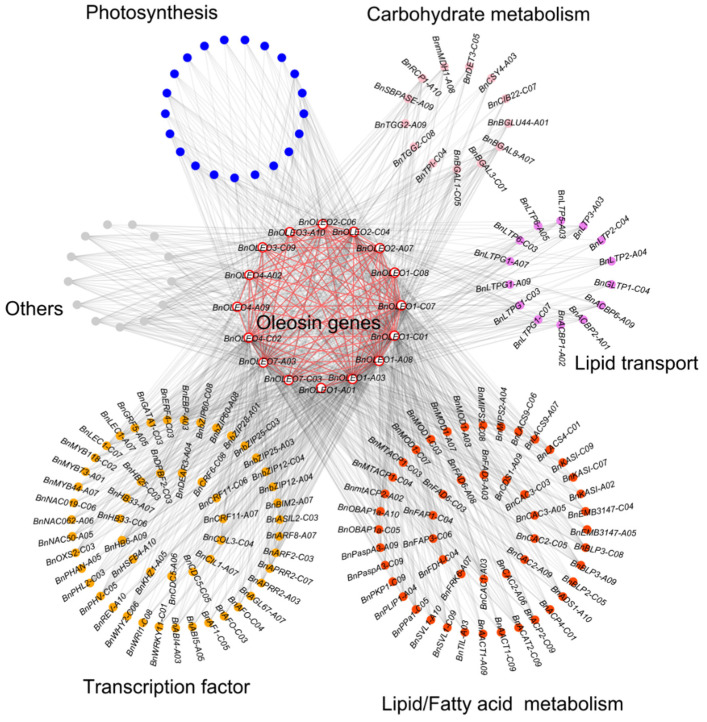
Co-expression network analysis of *BnOLEO* genes.

Based on functional annotation, the genes that were co-expressed with oleosin genes were further classified. 16 *BnOLEO* genes were directly linked to 165 genes, including 54, 50, 14, 21, and 13 genes related to TFs, lipid/fatty acid metabolism, lipid transport, photosynthesis, and carbohydrate metabolism, respectively (Figure 5; Table S9).

## 3. Discussion

In previous studies, when the main oleosin gene in Arabidopsis seeds was inhibited, the oil bodies became larger, with decreases in TAG accumulation levels [33,35]. Despite studies on genome-wide identification and functional analysis of oleosin genes in *B. napus* [29], the underlying molecular regulatory networks of the oleosin genes remain unclear. In this study, the promoters of 53 *BnOLEO* genes were studied for the presence of light-, hormone-, and stress-related cis-elements. Several types of light-responsive elements (for example, G-box, Box 4, and GT1-motif) and plant growth hormones, including abscisic acid, salicylic acid, and MeJA responsive elements, were identified in the candidate genes. Li et al. [36] suggested strong positive correlations between oil contents and different light intensities in *Brassica* species. Phytohormones play an important role in improving oil yield and quality by regulating several enzymatic activities and signaling responses [37]. Deletion of the G-box element was found to reduce the promoter activity for light and hormone responses [38]. Wu et al. [39] suggested that abscisic acid, salicylic acid, and MeJA at physiological concentrations played crucial roles in lipid accumulation. The occurrence of these cis-regulatory elements provided evidence of the role of *BnOLEO* genes in the accumulation of oil content in *B. napus*.

RNA-seq technology was used to investigate the expression patterns of oleosin genes at different stages of seed development among two HOC and two LOC inbred lines. In total, 16 oleosin genes in the SL and SH lineages were highly expressed during seed development, including *BnOLEO3-C09*, *BnOLEO4*-*A02*, *BnOLEO4*-*A09*, *BnOLEO2*-*C04*, *BnOLEO1*-*C01*, and *BnOLEO7*-*A03* in the HOC accessions, compared to the LOC accessions at 25, 35, and 45 DAP, in two different environments (Figure 3a). These results indicated that oleosin genes played an important role in the seed oil biosynthesis in *B. napus*. Meanwhile, a regional association analysis revealed that *BnOLEO1-C01*-Hap1 and *BnOLEO7-A03*-Hap1 corresponded to accessions with higher seed oil contents compared to other haplotype alleles (Figure 4, Figure S5, and Table S7). The haplotype region on the A03 chromosome, where *BnOLEO7-A03* is located, overlaps with a previously reported QTL [40]. Thus, these favorable haplotype alleles can be used to further improve SOCs in rapeseed.

In addition, co-expression network studies showed that 16 *BnOLEO* genes were directly linked to each other, as well as to TFs, including *LEC1*, *WRI1*, *WRKY1*, *bZIP12*, *bZIP25*, *bZIP28*, *bZIP60*, *MYB44*, *MYB73*, and *MYB118*. (Figure 5; Table S9). Prediction of the TF binding sites showed AP2/ERF, WRKY, bZIP, and MYB as the main gene families regulating oleosin genes (Figure 2, Table S3). TFs were found to be involved in positive or negative regulation of the expressions of genes responsible for oil content accumulation [41]. Over-expression of *LEC1* was reported to cause upregulated expressions of FA synthetic genes, and promoted oil content accumulation in *B. napus* [42]. *WRI1* was studied as a member of a plant-specific family of TFs (AP2/EREBP) involved in fatty acid production at the onset of the seed maturation phase in Arabidopsis [43]. *ABI4* was found to encode an AP2/ERF TF involved in lipid mobilization in Arabidopsis embryos [44]. A bZIP family TF, bZIP53, was shown to form a ternary complex with bZIP10 or bZIP25 and ABI3, and promoted the expressions of seed maturation genes and lipid accumulations [45]. Overexpression of *GmMYB73* was shown to enhance the lipid contents of transgenic Arabidopsis seeds and enhanced oil production in legume crop plants [46]. Barthole et al. [47] suggested that MYB118 repressing *LEC2* expressions influenced FA biosynthesis in Arabidopsis. Oleosin genes were found to be directly linked to FA synthesis (e.g., *CAC1*, *CAC2*, *CAC3*, *FAD3*, *KASI*, *MOD1*, *mtACP1*, and *mtACP2*) and lipid transfer (*ACBP1*, *ACBP2*, *ACBP6*, *LTP1*, *LTP2*, *LTP3*, *LTP5*, and *LTP6*) genes in the co-expression network. Bhatla et al. [48] suggested that the co-expression of oleosins and genes involved in TAG biosynthesis increased oil contents. These results suggested that oleosin genes, TFs, and lipid transport-related genes form a complex network that helps to co-regulate seed oil accumulation in *B. napus*.

## 4. Materials and Methods

### 4.1. Identification of Oleosin Genes in Arabidopsis and B. napus

Arabidopsis oleosin genes were searched for in The Arabidopsis Information Resource (TAIR) database (https://www.arabidopsis.org/ (accessed on 18 December 2021)) [49], using oleosin as the keyword query. HMMER3.2 software was used to search against the *B. napus* protein sequences using the HMM file of oleosin (PF01277) that was downloaded from the Pfam database (http://pfam.xfam.org/ (accessed on 1 January 2022)) [50]. The candidate *BnOLEO* proteins were obtained using *E*-values ≤ 1 × 10^−5^. The obtained Arabidopsis and *B. napus* oleosin sequences were confirmed using the NCBI Conserved Domain Search database [51]. When searching for sequence information, the numbers of amino acids, intron and exon numbers, coding sequence (CDS) lengths, and chromosomal locations of the oleosin genes were obtained from the CNS-Genoscope database [52].

### 4.2. Phylogenetic Analysis and Regulatory GENE Prediction of oleosin Genes

Multiple sequence alignments of all identified oleosin proteins in Arabidopsis and *B. napus* were conducted using ClustalW. A phylogenetic tree was constructed using the NJ phylogenetic method in MEGA7 [53] with 1000 bootstrap replicates. TBtools was used to obtain 2000 bp of the genomic DNA sequence located upstream of the start codon. The PlantCARE database (http://bioinformatics.psb.ugent.be/webtools/plantcare/html/ (accessed on 9 May 2022)) was used to predict the cis-acting elements in the promoter sequences (−2000 bp) [54]. PlantTFDB database (http://plantregmap.gao-lab.org/ (accessed on 10 May 2022)) was used to predict the TF binding sites in the promoter sequences, and the parameter was set to a *p* value ≤ 1 × 10^−6^ [55].

### 4.3. Gene Structure and Conserved Motif Analysis

The conserved motifs of oleosin were analyzed with MEME (https://meme-suite.org/meme/tools/meme (accessed on 11 March 2022)) [56], and the parameters were as follows: zoop (zero or one occurrence per sequence) was selected in site distribution, the width of motifs was 8 to 29, and the maximum number of motifs was set to 11. The exon–intron structures of the oleosin genes were analyzed by the Gene Structure Display Server 2.0 (http://gsds.gao-lab.org/ (accessed on 27 March 2022)) [57]. TBtools [58] was used to construct and merge the visualizations of all characteristic results.

### 4.4. Transcriptome Sequencing

Two HOC (ZY036 and ZY511) and two LOC (XY774-3 and XY15) Chinese semi-winter rapeseed inbred lines, obtained from Hunan Agricultural University in Changsha, China, were used for transcriptome profiling in our study. These four accessions were planted in Changsha (CS, 28.2282° N, 112.9388° E) and Kunming (KM, 25.0406° N, 102.7122° E). The total RNA of these four inbred lines was extracted from seeds using TRIzol reagent (Invitrogen, Carlsbad, CA, USA) at 25, 35, and 45 DAP, and were immediately frozen in liquid nitrogen and stored at −80 °C. The total RNA from each accession was used to construct paired-end sequencing libraries according to the manufacturer’s instructions (Illumina Inc., San Diego, CA, USA). Paired-end reads (125 bp) were evaluated using the Illumina HiSeq 2500 platform. Next, the raw data were preprocessed using the Illumina paired-end RNA-seq approach to remove adapter sequences, low-quality sequences (average quality scores less than 20), short sequences (lengths less than 100), and poly-A tails before assembly. Finally, high-quality sequencing data for each accession were generated.

### 4.5. Expression Analysis of Oleosin Genes

The high-quality reads were subsequently aligned to the Darmor-bzh reference genome v.4.1 [52] using HISAT2. HTSeq v.0.6.1 [59] was used to estimate the gene expression levels in the four accessions. The fragments per kilobase of transcript per million fragments mapped (FPKM) of each gene were then calculated based on gene length and read counts [60]. A heatmap drawn by Heatmap Illustrator 1.0 (Hemi 1.0) was used to visualize the similarities and differences of the oleosin genes [61]. qRT-PCR was carried out to confirm the RNA-seq results. First-strand cDNA was synthesized using PrimeScript RT Master Mix (TaKaRa Biotech, Dalian, China) according to the manufacturer’s instructions. Analysis of the results was performed using LightCycler 480 SYBR Green I Master Mix and a LightCycler 480II real-time PCR system (Roche, Switzerland). *BnEF* was selected as the reference gene [62,63]. Three biological replicates and three measurements for each replicate were performed under identical conditions. The 2^−ΔΔCT^ method was used to estimate the relative expression levels [64]. The gene-specific primer sequences are listed in Table S10.

### 4.6. Regional Association Analysis

The re-sequencing of 50 rapeseed accessions was described in detail by Dong et al. [65], including removal of SNP loci with heterozygous rates > 0.25 and MAFs < 0.05. A total of 532,005 high-quality SNP markers were used to determine the correlations among oleosin genes and oil contents in 50 re-sequenced rapeseed inbred lines. Yao et al. [20] provided a detailed description of the population structure and a principal component analysis (PCA) of 50 Chinese semi-winter rapeseed accessions. Marker–trait associations were identified by TASSEL v.5.0 software [66] with a mixed linear model (MLM) [67]. Manhattan plots were constructed using the R package “qqman” [68]. In order to calculate the FDR threshold with the R package fdrtool [69], we estimated the significant associations between SNPs and seed oil content phenotypes using a threshold value of −log_10_^(*P*)^ = 4.0.

### 4.7. Co-Expression Network

WGCNA is an effective way to identify clusters of highly correlated genes. The “WGCNA” R package [70] was used to construct co-expression networks with a cutoff value of 0.15 for the weight parameter. Visualization of the co-expression network was performed using Cytoscape v.3.6 [71]. GO enrichment analysis was completed by TBtools [58] and visualized using the R package, “ggplot2” [72].

## 5. Conclusions

The differentially expressed oleosin genes among two HOC and two LOC accessions were explored in *B. napus*. *BnOLEO3-C09*, *BnOLEO4-A02*, *BnOLEO4-A09*, *BnOLEO2-C04*, *BnOLEO1-C01,* and *BnOLEO7-A03* showed higher expressions in the HOC accessions compared to the LOC accessions, at 25, 35, and 45 DAP, in two different environments. A regional association analysis revealed that the natural variations in the *BnOLEO1-C01* and *BnOLEO7-A03* gene regions were related to phenotypic variations in the oil content of 50 re-sequenced rapeseed accessions. A co-expression network analysis showed that the oleosin genes were directly related to lipid/fatty acid metabolism, TFs, and lipid transport- and carbohydrate-related genes, thus forming a molecular network involved in the potential regulation of seed oil accumulation. This study holds great significance for the development of oleosin gene-specific markers and molecular marker-assisted selection breeding.

## Data Availability

The data sets supporting the results of this article are included within the article and its additional files.

## Supplementary Materials

The following supporting information can be downloaded at: https://www.mdpi.com/article/10.3390/plants11223140/s1, Figure S1: Phylogenetic tree, gene structure, and gene motifs of oleosin of A. thaliana and B. napus; Figure S2: The average expression levels of Arabidopsis oleosin genes in developmental map; Figure S3: The average expression levels of Arabidopsis oleosin genes in developmental map; Figure S4: Expression patterns of oleosin genes in different stages of seed development after pollination in B. napus; Figure S5: Regional association analysis of seed oil contents of chromosome A03 with whole-genome resequencing of 50 accessions; Figure S6: Gene co-expression network construction; Figure S7: GO enrichment analyses of target module genes; Table S1: List of oleosin genes identified in Arabidopsis and B. napus; Table S2: The cis-elements on the promoter regions of oleosin genes in B. napus. Table S3: Transcription factor binding sites in the promoter region of oleosin genes in B. napus. Table S4: Number of transcription factors with potential binding sites in promoter regions of oleosin genes; Table S5: The average expression levels of Arabidopsis oleosin genes in developmental map; Table S6: The FPKM value of oleosin genes at different stages of seed development; Table S7: Detailed analysis of haplotype alleles of oleosin genes related to oil content in the haplotype regions of chromosome C01 and A03; Table S8: GO enrichment analyses of target module genes; Table S9: List of genes and their corresponding FPKM values in the co-expression subnetwork; Table S10: Primers used for qRT-PCR.
