# Supplementary material for: Transcriptome and Regional Association Analyses Reveal the Effects of Oleosin Genes on the Accumulation of Oil Content in Brassica napus
Source: Plants (Basel). 2022 Nov 16;11(22):3140. doi: 10.3390/plants11223140 (PMC9698637; doi:10.3390/plants11223140)
Supplement: Supplementary file 1 [file plants-11-03140-s001.zip › Supplementary Figures.pdf]

Supplementary Figures

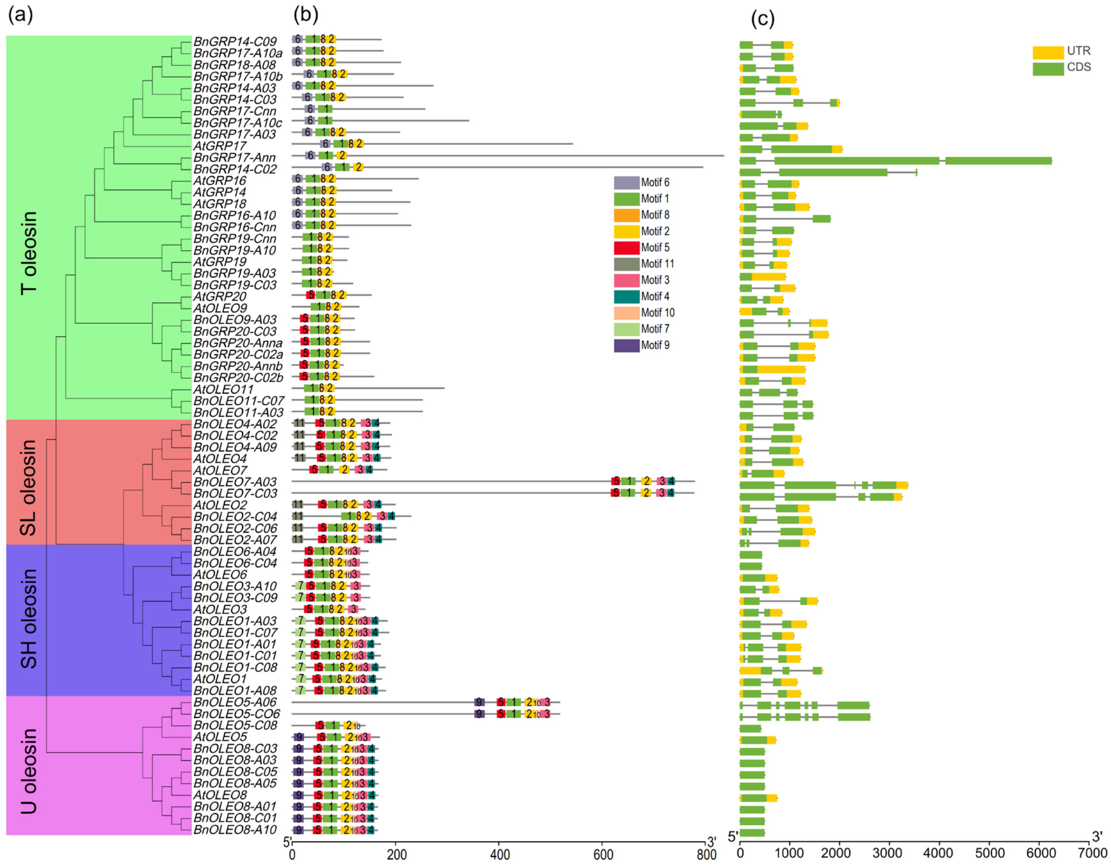

Figure S1. Phylogenetic tree, gene structure, and gene motifs of oleosin of *A. thaliana* and *B. napus*. (a) The neighbor-joining tree was generated from the alignment of oleosin genes from Arabidopsis and *B. napus*. The different background colors represent the four lineages. (b) Conserved motifs were detected using MEME and were shown as boxes of different colors (numbered as motifs 1–11). (c) Green boxes, black lines, and yellow boxes indicate CDS, introns, and untranslated regions (UTRs) of genes, respectively.

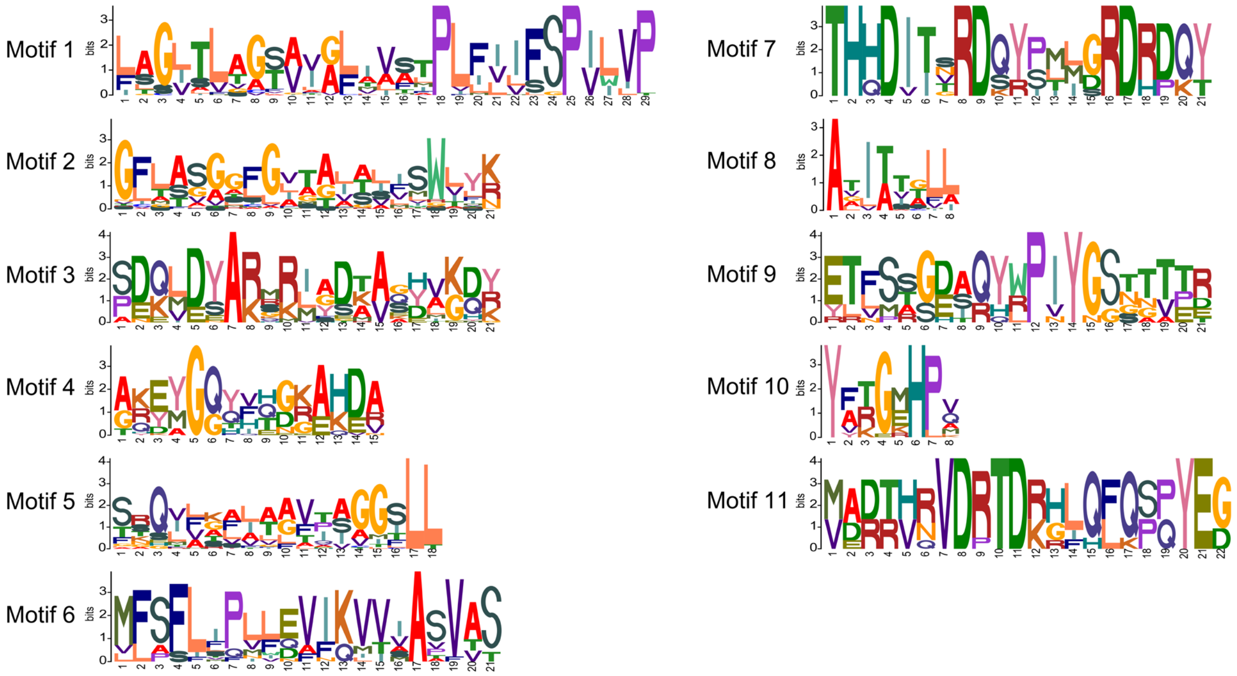

Figure S2. Informations of motifs in oleosin genes family.

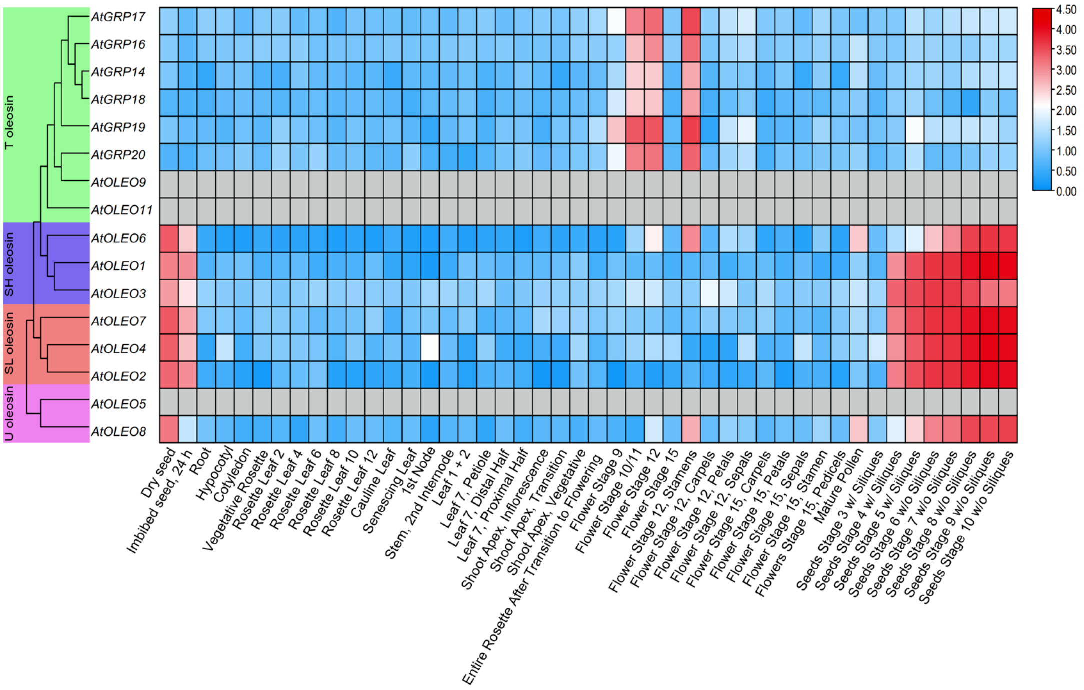

Figure S3. The average expression levels of Arabidopsis oleosin genes in developmental map. The color bar shows the gene expression with $\log_{10}{}^{(FPKM + 1)}$. Red indicate high expression levels, blue indicate low expression levels and grey indicates no expression levels.

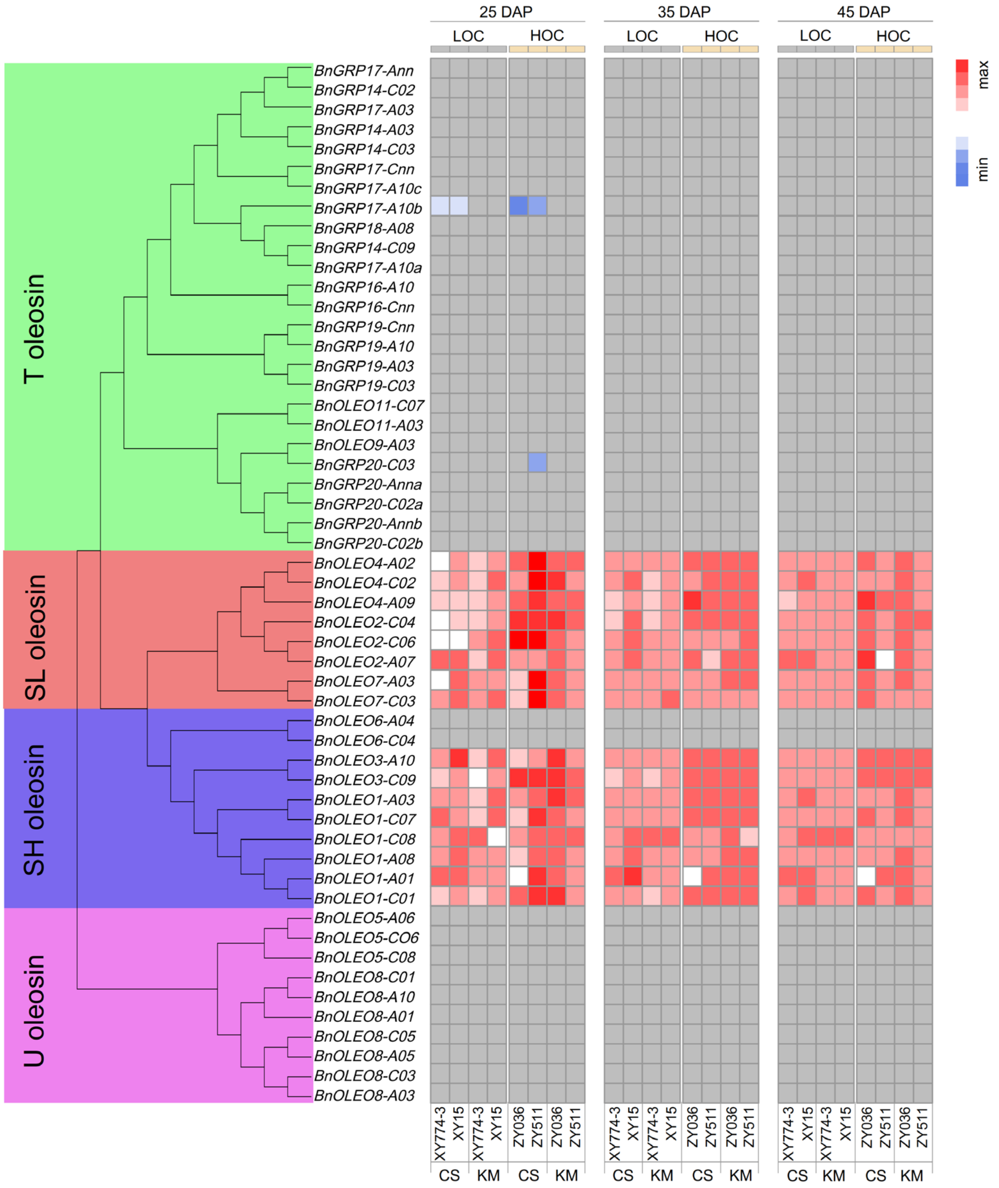

Figure S4. Expression patterns of oleosin genes in different stages of seed development after pollination in *B. napus*. The heatmap is generated by comparing the relative results of different genes in different periods according to the log_{10} transformed values of FPKM. Red indicate high expression levels and grey indicates no expression (FPKM = 0). LOC, low-oil content; HOC, high-oil content; CS, Changsha; KM, Kunming.

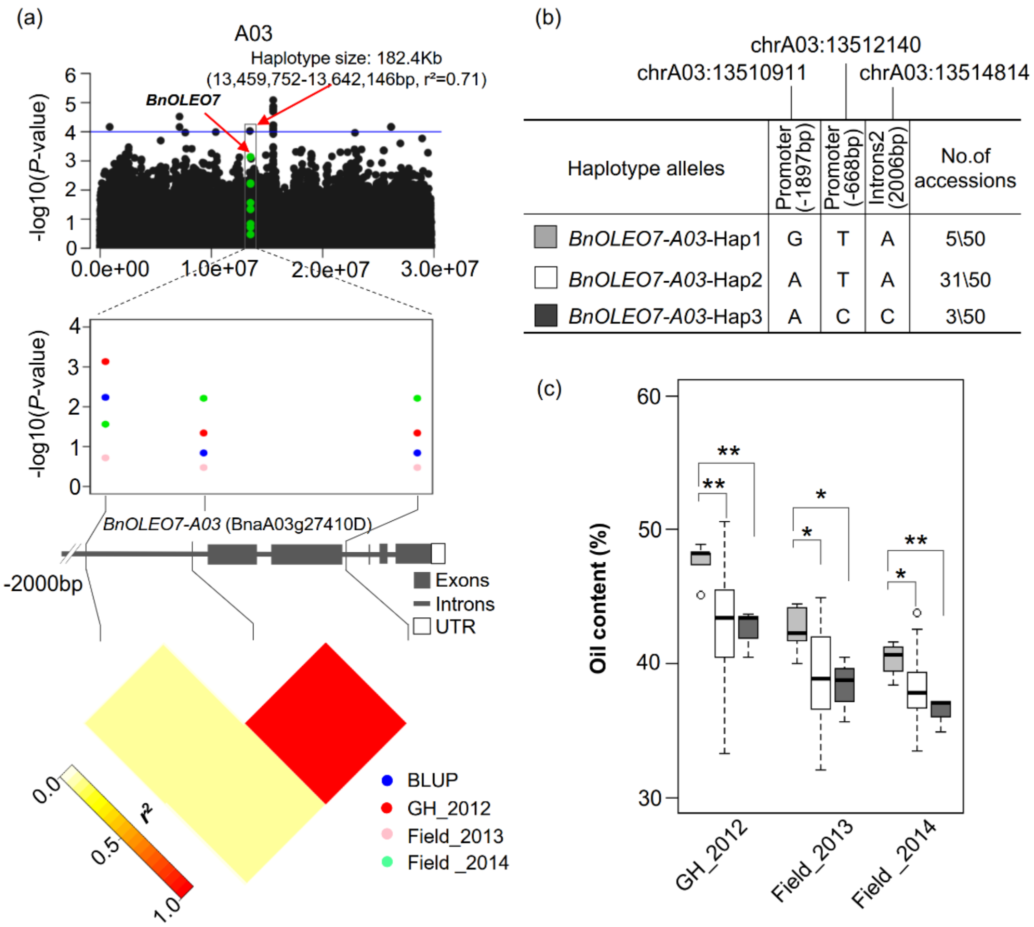

Figure S5. Regional association analysis of seed oil contents of chromosome A03 with whole-genome resequencing of 50 accessions. (a) Haplotype region (13,459,752–13,642,146 bp, $r^2 = 0.71$) was significantly associated with seed oil content. The chrA03:13,510,911, chrA03:13,512,140, and chrA03:13,514,814 SNPs are located in the promoter and Introns 2 of *BnOLEO7-A03* (BnaA03g27410D) gene in the haplotype region, respectively. The heatmap shows that these SNPs have a strong LD. (b) Three haplotype alleles with frequencies greater than 0.01 were identified in *BnOLEO7-A03* haplotype region. (c) The boxplots show that *BnOLEO7-A03*-Hap1 has a higher seed the oil contents than the other two haplotype groups. The symbols indicate significant differences in the haplotype groups compared with Hap1. *: $p \leq 0.05$, **: $p \leq 0.01$.

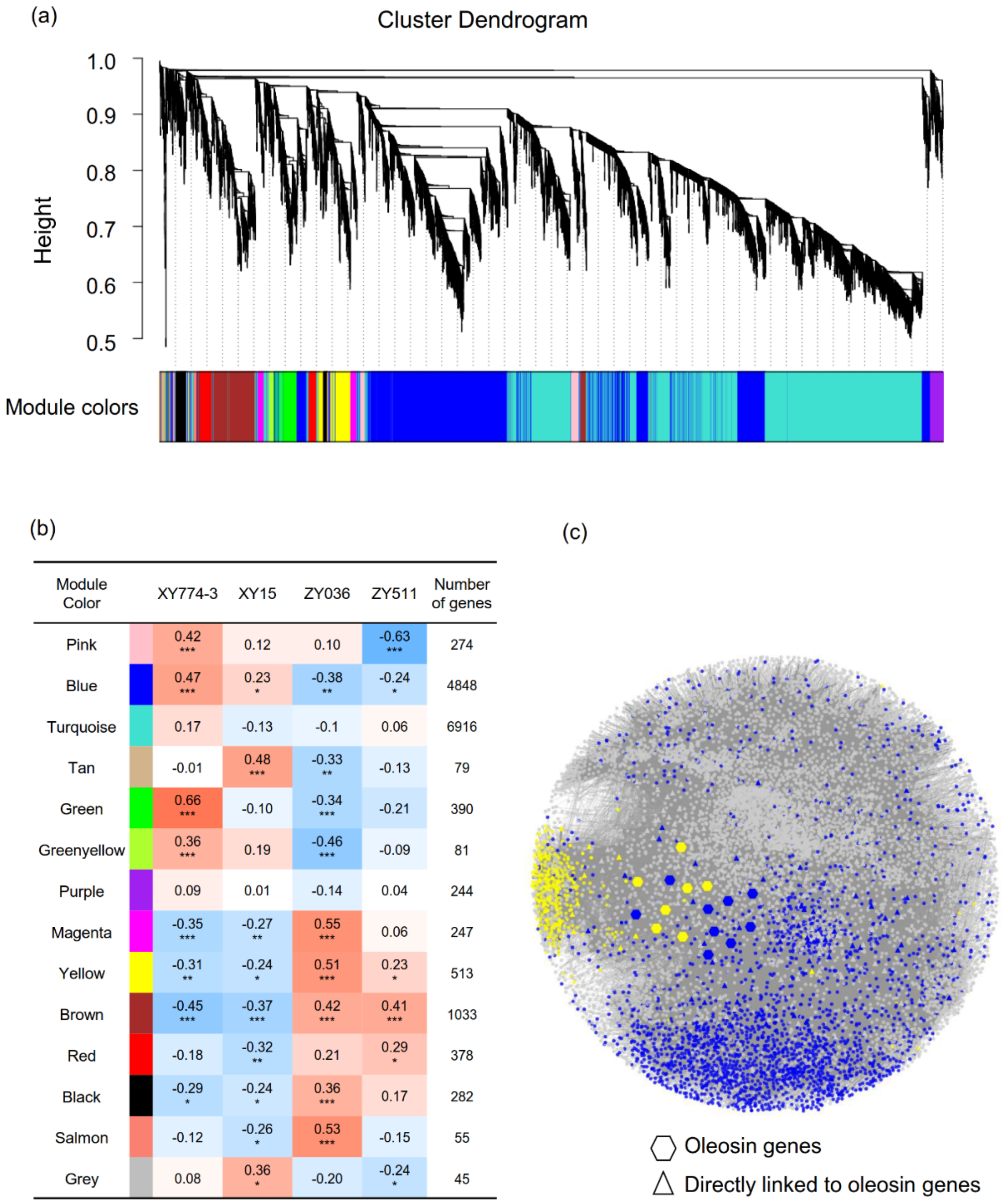

Module Color	XY774-3	XY15	ZY036	ZY511	Number of genes
Pink	0.42 ***	0.12	0.10	-0.63 ***	274
Blue	0.47 ***	0.23 *	-0.38 **	-0.24 *	4848
Turquoise	0.17	-0.13	-0.1	0.06	6916
Tan	-0.01	0.48 ***	-0.33 **	-0.13	79
Green	0.66 ***	-0.10	-0.34 ***	-0.21	390
Greenyellow	0.36 ***	0.19	-0.46 ***	-0.09	81
Purple	0.09	0.01	-0.14	0.04	244
Magenta	-0.35 ***	-0.27 **	0.55 ***	0.06	247
Yellow	-0.31 **	-0.24 *	0.51 ***	0.23 *	513
Brown	-0.45 ***	-0.37 ***	0.42 ***	0.41 ***	1033
Red	-0.18	-0.32 **	0.21	0.29 *	378
Black	-0.29 *	-0.24 *	0.36 ***	0.17	282
Salmon	-0.12	-0.26 *	0.53 ***	-0.15	55
Grey	0.08	0.36 *	-0.20	-0.24 *	45

Figure S6. Gene co-expression network construction. (a) Cluster dendrogram showing co-expression modules. Each leaf in the tree is one gene and modules corresponding to branches are labeled by different colors. (b) Heatmap of module-sample association depicting correlations between modules and the high-low oil content. Each row corresponds to a module. The correlation coefficient and the corresponding p-value are shown in the matrix. (c) Co-expression network of oleosin genes in the seed of *B. napus*. Hexagon represent oleosin genes, triangle represent these genes directly linked with oleosin genes. Only the color of the module where the oleosin genes are located are shown in the figure, which is blue and yellow, respectively.

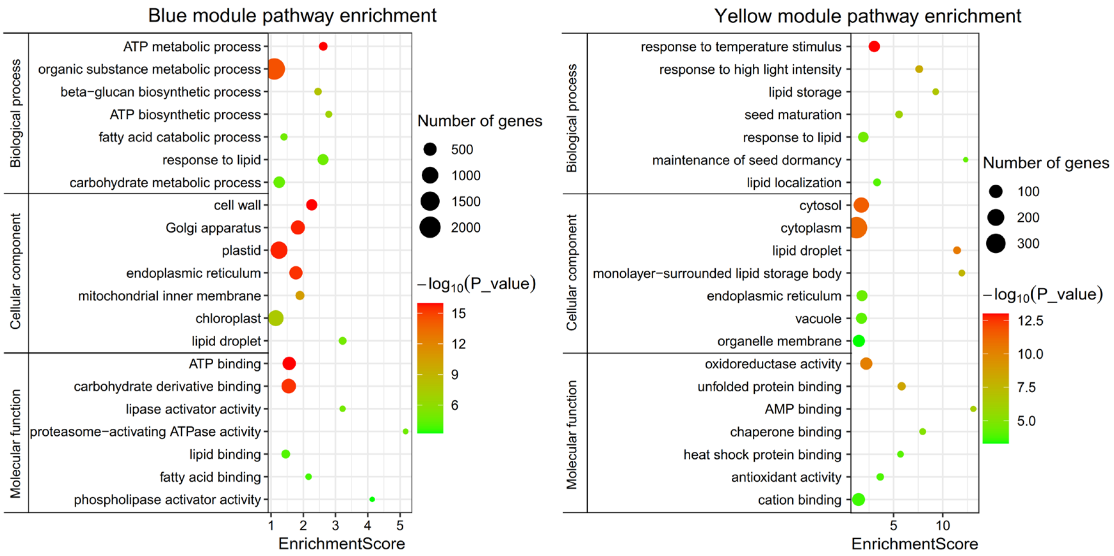

Figure S7. GO enrichment analyses of target module genes.
